# Whole-Genome Sequence of *Streptococcus parauberis* Strain SP-llh, Isolated from Cows with Mastitis in Western China

**DOI:** 10.1128/genomeA.01389-16

**Published:** 2017-01-12

**Authors:** Longhai Liu, Feng Yang, Xinpu Li, Jinyin Luo, Zhe Zhang, Hongsheng Li

**Affiliations:** Lanzhou Institute of Husbandry and Pharmaceutical Sciences of Chinese Academy of Agricultural Sciences, Lanzhou, Gansu, People's Republic of China

## Abstract

*Streptococcus parauberis* strain SP-llh was isolated from cows with mastitis in western China in 2015. The 2,522,235-bp genome sequence consists of 46 large contigs in 14 scaffolds and contains 2,620 predicted protein-coding genes, with a G+C content of 35.3%.

## GENOME ANNOUNCEMENT

*Streptococcus parauberis* is a coccoid, nonmotile, alpha-hemolytic, and Gram-positive bacterium of the *Streptococcaceae* family, and it belonged to *Streptococcus uberis* previously. Williams and Collins proposed *Streptococcus parauberis* instead of *Streptococcus uberis* type II based on phylogenetically distinct nucleotide sequences of 16S rRNA of *Streptococcus uberis* types I and II in 1990 (1). *Streptococcus parauberis* is a cause of bovine mastitis and fish infections. There have been few documents about the complete genome sequences of bovine *Streptococcus parauberis*. Now, we report the whole-genome sequence of *Streptococcus parauberis* strain SP-llh in order to study the characteristics of a bovine *Streptococcus parauberis* strain.

We identified the strain by biochemical and PCR assays. The genomic DNA of SP-llh strain was extracted using an SDS method. Total DNA obtained was subjected to quality control by agarose gel electrophoresis and quantified by Qubit. The genome of SP-llh strain was sequenced with massively parallel sequencing (MPS) Illumina technology. We constructed two DNA libraries: a paired-end library with an insert size of 500 bp, and a mate-pair library with an insert size of 5 kb. The 500-bp library and the 5-kb library were sequenced using an Illumina HiSeq 2500 by PE125 strategy. Library construction and sequencing were performed at the Beijing Novogene Bioinformatics Technology Co., Ltd. Quality control of both paired-end and mate-pair reads was performed using an in-house program. After this step, Illumina PCR adapter reads and low-quality reads were filtered. The filtered reads were assembled by SOAPdenovo (2, 3) to generate 46 contings, and the assembly falls to 14 scaffolds.

Transfer RNA (tRNA) genes, ribosomal RNA (rRNA) genes, and small RNAs (sRNAs) were predicted with tRNAscan-SE (4), rRNAmmer (5), and BLAST against the Rfam (6) database, respectively, yielding 31 tRNA genes, two rRNA operons, and six sRNA. Ten repetitive sequences were predicted using RepeatMasker (7), and six tandem repeats were analyzed using Tandem Repeat Finder (8). Furthermore, prophage and clustered regularly interspaced short palindromic repeats (CRISPRs) were predicted using PHAST (9) and CRISPRFinder (10), respectively.

Gene prediction was performed on the strain SP-llh genome assembly by GeneMarkS (11) with integrated model which combines the GeneMarkS generated (native) and Heuristic model parameters. A whole-genome BLAST (12) search (*E* value less than 1e-5, minimal alignment length percentage larger than 40%) was performed against thge Kyoto Encyclopedia of Genes and Genomes (KEGG) (13–15), Clusters of Orthologous Groups (COG) (16, 17), Nonredundant Protein (NR), Swiss-Prot (18), Gene Ontology (GO) (19), and TrEMBL (18) databases.

The *Streptococcus parauberis* genome includes 2,522,235 bp and is composed of 2,620 predicted coding sequences (CDSs), with a G+C content of 35.3%. 

### Accession number(s).

The genome sequence of *Streptococcus parauberis* SP-llh is available in GenBank under the accession number MLCP00000000. The version described in this paper is version MLCP00000000.1.
